# Master clinical medical knowledge at certificated-doctor-level with deep learning model

**DOI:** 10.1038/s41467-018-06799-6

**Published:** 2018-10-19

**Authors:** Ji Wu, Xien Liu, Xiao Zhang, Zhiyang He, Ping Lv

**Affiliations:** 1 Department of Electronic Engineering, Tsinghua University, Beijing, 100084 China; 2 Medical Business Department, iFlytek Co.Ltd, Hefei, 230088 China; 3 Tsinghua-iFlytek Joint Laboratory, iFlytek Research, Beijing, 100084 China

## Abstract

Mastering of medical knowledge to human is a lengthy process that typically involves several years of school study and residency training. Recently, deep learning algorithms have shown potential in solving medical problems. Here we demonstrate mastering clinical medical knowledge at certificated-doctor-level via a deep learning framework Med3R, which utilizes a human-like learning and reasoning process. Med3R becomes the first AI system that has successfully passed the written test of National Medical Licensing Examination in China 2017 with 456 scores, surpassing 96.3% human examinees. Med3R is further applied for providing aided clinical diagnosis service based on real electronic medical records. Compared to human experts and competitive baselines, our system can provide more accurate and consistent clinical diagnosis results. Med3R provides a potential possibility to alleviate the severe shortage of qualified doctors in countries and small cities of China by providing computer-aided medical care and health services for patients.

## Abstract (web summary)

AI is used increasingly in medical diagnostics. Here, the authors present a deep learning model that masters medical knowledge, demonstrated by it having passed the written test of the 2017 National Medical Licensing Examination in China, and can provide help with clinical diagnosis based on electronic health care records.

## Introduction

Qualified medical practitioners are in severe shortage in many countries of the world and medical training is typically a lengthy procedure. For example, a medical student usually spends more than 5 years of school study and then takes a few years of residency training. Though in recent years, plenty of medical AI algorithms/systems spring up in both the research and the industry communities, almost all of them are designed to merely solve some pre-specified medical problems, such as classifying skin cancer, detecting pneumonia, and producing treatments for a few pre-defined cancers or diseases. There is still lack of an efficient AI-enabled computer model which, like candidate general practitioners, can automatically learn and master a wide range of medical knowledge from a large medical corpus, and apply medical knowledge, concepts, and principles to solving generic medical problems. The barriers for achieving this goal mainly include (1) learning such wide range of medical knowledge from text corpus is still an unsolved challenging problem in research communities; (2) understanding medical problems and making reasoning with medical-views at human-doctor-level is also very difficult for a computer program.

In this study, we propose a novel deep learning model Med3R (Free **R**eading, Guided **R**eading and Multi-layer **R**easoning) to solve these problems. The proposed model employs a human-like learning and reasoning framework that firstly captures primary medical knowledge from a large medical corpus with a “Free Reading” module, then masters more precise knowledge via a “Guided Reading” phase, and ultimately makes inference/decision in a “Multi-layer Reasoning” fashion. Med3R were examined by taking the written test of National Medical Licensing Examination in China 2017. The results officially reported by National Medical Examination Center (NMEC)^1^show that Med3R has successfully passed the exam and surpassed 96.3% human examinees. Med3R also can be applied for providing aided clinical diagnosis service and the experimental results illustrate that the model can provide more accurate and consistent results compared to human experts and competitive baselines. Our study shows that deep learning techniques have potential abilities to master medical knowledge and provide accurate clinical diagnosis suggestions based on medical electronic records and that it provides a possibility to alleviate the severe shortage of qualified doctors in countries and small cities of the world.

## Results

### Lab results

**Fig. 1:**
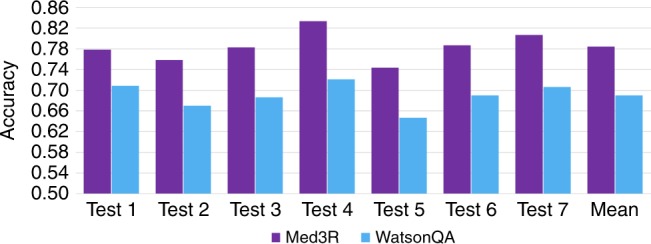
Comparison results of Med3R with WatsonQA on 7 practice tests. From left to right, “Test 1” means the comparison accuracy on the first practice test, and “Test 2” means the accuracy on the second practice test, and so on. The last column indicated by “Mean” is the average accuracy over the 7 practice tests

Before officially taking NMLEC 2017, we employed medical experts to produce 7 practice tests to evaluate and analyze our Med3R system. Each of the 7 practice tests strictly satisfies all the requirements of NMLEC, such as the number of questions, question type, distribution of difficulty, and the coverage of medical knowledge etc. A comparison of results of our proposed Med3R system with a WatsonQA-alike system is presented in Fig. 1. The results illustrate that our Med3R system obtained an average accuracy of 0.78 over the 7 practice tests. The average accuracy is higher than that of a WatsonQA system and is also much higher than expected successful-passing level (accuracy of 0.6). To analyze the performance of our layered reasoning module, we conducted a series of comparison experiments of which each only uses one of the following reasoning layers: keypoint reasoning (KR), context reasoning (CR) and global reasoning (GR). The results, presented in Table 1, demonstrate that only using one layer of reasoning can achieve a relatively satisfying average accuracy of 0.67–0.68 (>0.6), but with three reasoning layers used simultaneously an improved result (an average accuracy of 0.78) can be obtained. It indicates that the proposed three reasoning layers are strongly complementary. Additionally, we also compared our proposed method to several modern deep learning based reasoning models, such as r-net^2^, neural reasoner^3^, and iterative attention^4^. The results illustrate that the performance of our proposed reasoning methods is also superior to these competitive baselines.

**Table 1:** Comparison of different reasoning methods

Reasoning Method	Test 1	Test 2	Test 3	Test 4	Test 5	Test 6	Test 7	Mean
Med3R	0.78	0.76	0.78	0.83	0.74	0.79	0.81	0.78
Med3R (KR only)	0.70	0.63	0.66	0.70	0.64	0.69	0.67	0.67
Med3R (CR only)	0.68	0.63	0.68	0.72	0.68	0.69	0.70	0.67
Med3R (GR only)	0.69	0.64	0.69	0.73	0.66	0.66	0.69	0.68
Iterative Attention^4^	0.62	0.54	0.61	0.65	0.57	0.64	0.59	0.61
Neural Reasoner^3^	0.50	0.48	0.49	0.52	0.52	0.53	0.52	0.50
R-net^2^	0.51	0.49	0.54	0.54	0.54	0.54	0.55	0.52

Note: KR only: only using Keypoint Reasoning, CR only: only using Context Reasoning, GR only: only using Global Reasoning

### NMLEC 2017 results

**Fig. 2:**
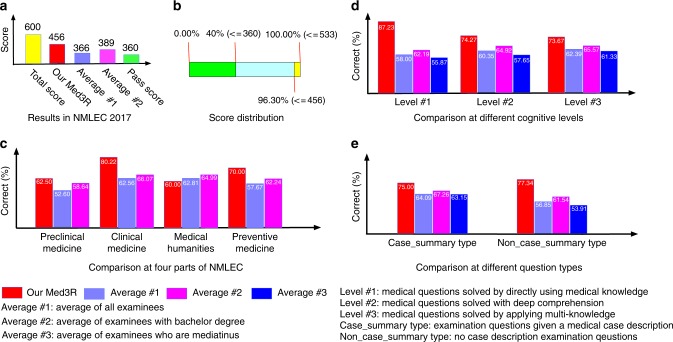
The results of Med3R system participating in the written test of NMLEC 2017. Our Med3R achieved **a** 456 scores much higher than the passing-requirement of 360 scores, and **b** surpassed 96.3% human examinees. The questions of NMLEC 2017 are categorized by medical experts into different subgroups to further analyze our system’s performance. For example, the comparison at **c** 4 different subjects, **d** three different cognitive levels, and **e** two different types of questions. All results and data in this figure are officially offered by NMEC^1^

Our Med3R was officially entitled by National Health Commission of the People’s Republic of China (NHCPRC)^5^ as a special “examinee” to take the written test of NMLEC (Supplementary Figs. 1, 2) during Aug 26–27, 2017. According to the examination result report (Supplementary Fig. 3) officially offered by NMEC^1^, the Med3R system successfully passed the exam with 456 scores (the passing score is 360). Results, presented in Fig. 2 and Supplementary Fig. 4, show that our system has excellent reasoning abilities for solving medical questions and surpasses 96.3% human examinees. We notice that the performance (accuracy 0.76 = 456/600) in NMLEC 2017 is very close to the performance (average accuracy of 0.78) in our testing on 7 practice tests (Fig. 1 and Table 1). To have a sense of the difficulty of NMLEC 2017 and the generalization ability of our model, we calculated the similarity degrees (Supplementary Methods) between questions from NMLEC 2017 and questions from our training dataset MedQA (more details about the dataset see Supplementary Methods) with Levenshtein distance^6^, and the results (Supplementary Fig. 5) show that there exist very few questions having strong textual similarity to questions in the training dataset.

### Real-world results

**Fig. 3:**
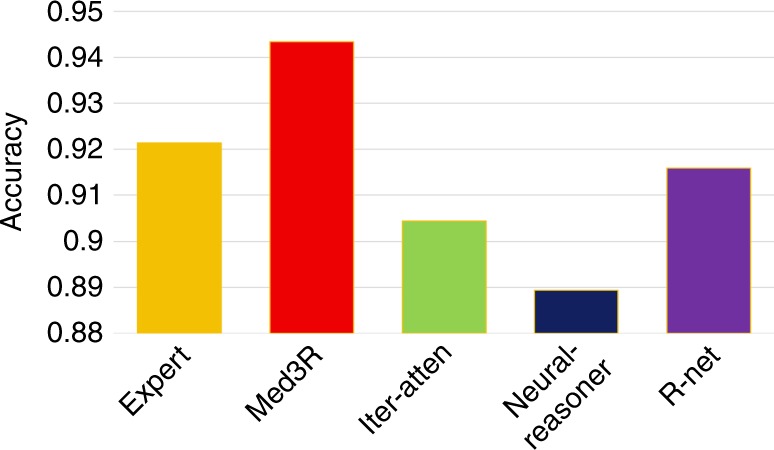
Comparison results of Med3R with baselines on clinical diagnosis. Med3R: our proposed NMLEC system fine-tuned on EMR data; expert: average accuracy of four medical experts; Iter-atten: iterative attention model^4^; Neural-reasoner: neural reasoner model^3^; and R-net: r-net model^2^. All baseline models are trained on the same data as used in Med3R model

**Fig. 4:**
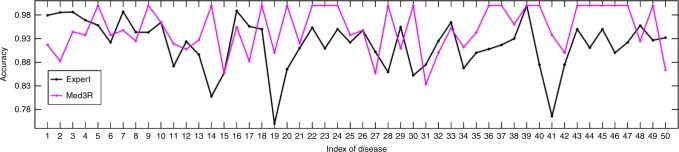
Accuracy comparison between the Med3R system and medical experts. The accuracy of expert listed here is the average accuracy of four medical experts (Details about the annotation accuracy of four medical experts is given in the Supplementary Fig. 10) over 50 diseases (see Supplementary Table 3)

Medical health service and healthcare is severely unbalanced nation-wide in China. For example, countries and small cities of China suffer from the long-term shortage of qualified doctors. Our proposed framework Med3R provides a potential possibility for providing medical cares and health services using computer models. After successfully passing NMLEC 2017, the model was applied for aided clinical diagnosis in several trial areas of China, such as Hefei Luyang District. To evaluate our system’s performance, we conducted a comparison with human experts. 5000 samples of Electronic Medical Record (EMR) are collected from the trial areas and annotated by four board-certified clinicians (Supplementary Table 1). The collected EMR sample (Supplementary Fig. 6) mainly consists of three parts: (1) chief complaint, (2) history of present illness, and (3) disease code. For each sample, chief complaint and history of present illness are concatenated as the input of our system and disease code is the label. The tested 5,000 EMR samples refer to 50 diseases (Supplementary Table 3), 100 samples for each disease. When the NMLEC trained model is directly used on EMR dataset for providing diagnosis, we observed a performance (totally accuracy of 92.04%) comparable to human experts (Supplementary Fig. 7). As we know, the writing style of EMRs data is different from that of NMLEC. We used another 10,000 EMR samples for a further adaption (fine-tuning) of the Med3R model. The comparison results (also tested on the annotated 5000 samples), presented in Fig. 3, show that the fine-tuned Med3R system surpassed medical experts’ level at mean accuracy on diagnosis results over the tested 50 diseases. The comparison results of each tested disease, presented in Fig. 4, illustrate that our system is more robust and consistent than human experts. More details about the accuracy of the four medical experts over the tested 50 diseases are given in Supplementary Fig. 8.

## Discussion

In this work we have developed a deep learning model, based on a two-stage representation learning module “Free Reading” and “Guided Reading” and a reasoning module “Multi-layer Reasoning”, that has surpassed the vast majority of human examinees (96.3%) in the written test of NMLEC 2017 (an essential qualifying examination for being certified doctors in China) and also achieved higher accuracy than human experts on the clinical diagnosis test based on real EMR data. This study sheds substantial light on mastering clinical medical knowledge by using deep learning techniques. We have extended the modern embedding learning techniques into a more effective representation learning schema, by combining the manners of unsupervised learning and supervised learning, for acquiring medical knowledge from a large semi-structured medical corpus. We have presented a new reasoning module for answering medical questions or giving clinical diagnoses with a multi-scale fashion that combines the merits of reasoning at some key points, at a salient sentence, and at the whole supporting evidence material. This reasoning module can achieve robust and consistent performance in the medical examination test and the real clinical diagnostic test. Though there is a very long way to build an AI-enabled system which can deal with all kinds of medical problems as human medical experts, the work presented in this paper provides a potential possibility to improve medical conditions for medically underserved areas by providing computer-aided diagnosis suggestions or medical care services for patients.

## Methods

### Med3R

**Fig. 5:**
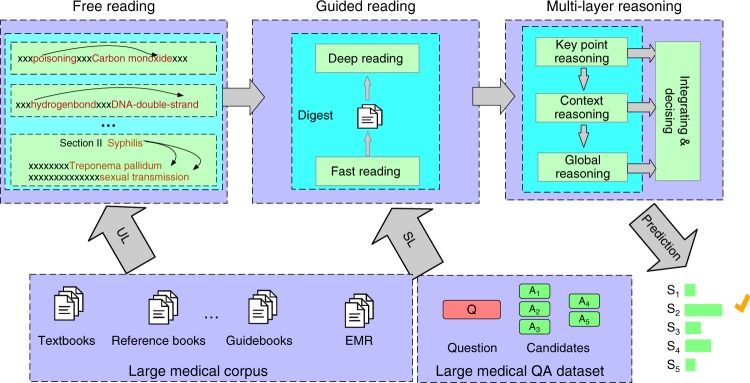
Architecture of our proposed deep learning framework Med3R. Med3R is consists of three modules: Free **R**eading, Guided **R**eading, and Multi-layer **R**easoning. Firstly, primary medical knowledge is coarsely captured from medical text via the “Free Reading” module which is trained over large medical corpus with Unsupervised Learning (UL) methods. Then, Supervised Learning (SL) methods are conducted in the “Guided Reading” module where a “Fast Reading” strategy is first used to collect a small digest (strongly related with given medical questions) from large medical corpus, then “Deep Reading” strategies are employed to analyze the digest and the given medical questions in deep manners. In the Reasoning phase, the “Multi-layer Reasoning” module is used to produce robust decision-makings by integrating reasoning at key-points level (“KeyPoint Reasoning”), sentence-context level (“Context Reasoning”) and global-digest level (“Global Reasoning”), respectively

The whole framework of Med3R is presented in Fig. 5, which consists of three parts,“Free Reading”, “Guided Reading”, and “Multi-layer Reasoning”. The parts of “Free Reading” and “Guided Reading” play a medical knowledge representation learning role, and the “Multi-layer Reasoning” is a reasoning module for making inference for medical questions or clinical diagnosis. In the rest of this section, we will introduce these modules in detail.

### Free reading and guided reading

Building knowledge graph via using triple (entity_*a*, relation, entity_*b*) as the basic element is a popular manner for representing knowledge^7–9^. But, this manner is very labor-intensive and time-consuming. Though there are some techniques aiming for automatically extracting entities and relations^10–16^, building a usable knowledge graph without mass human labor is still impossible. More importantly, this manner of knowledge representation lack feasibility and flexibility for building modern machine learning models, especially involving deep learning algorithms. Besides the explicit knowledge representation methods, implicitly embedding knowledge into continuous vectors, also called embeddings^17–19^, is another potential candidate. However, the commonly used word embeddings only depict the dependency of local context; thus they are promoted to capture common shallow semantic information^20^, but insufficient to discover rich medical knowledge hidden in large medical corpus^21^. Here, we generalize the implicit knowledge learning and representing method into a two-step fashion: “Free Reading” followed by “Guided Reading”.

**Fig. 6:**
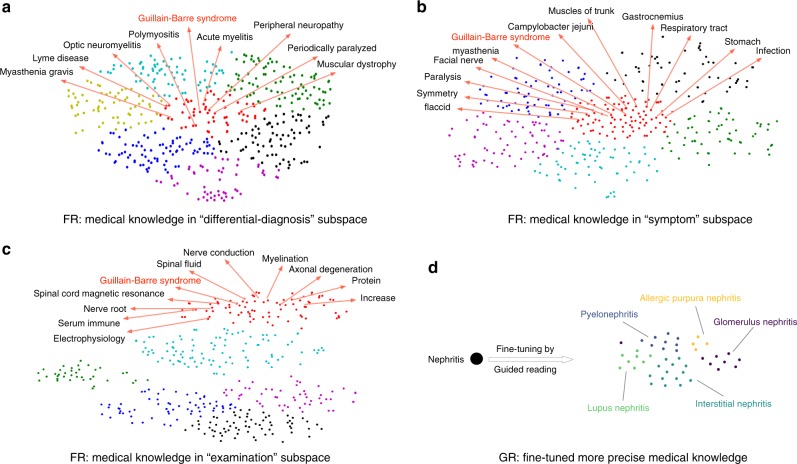
Example of medical knowledge obtained with “Free Reading” and “Guided Reading**”**. We use implicit embeddings to represent and capture medical knowledge from a large medical corpus. In the FR phase, we produce a series of embeddings to depict different kinds of medical knowledge. Take disease “Guillain-Barre syndrome” for example, in **a** “differential-diagnosis” embedding subspace similar diseases are close to each other, while in **b** “symptom” embedding subspace medical terms describing symptom of Guillain-Barre syndrome are clustering, and while in **c** “examination” embedding subspace medical terms related to examination to “Guillain-Barre syndrome” are clustering. In the GR phase, medical terms’ embeddings obtained from FR are fine-tuned by supervised learning models to get more rich and precise medical knowledge based on their context. For example, in **d** the embedding of “nephritis” is fine-tuned into different variants to describe and represent subtle different meanings in different context

In the “Free Reading” phase, a series of unsupervised learning algorithms are trained over large medical corpus to produce various kinds of embeddings. The generalized embedding learning schema can be described by

1 $${\mathrm{max}}\mathop {\sum}\limits_{ < s_{\mathrm{a}},s_{\mathrm{b}} > \in R_i} P(s_{\mathrm{b}}|s_{\mathrm{a}}) = \begin{array}{*{20}{c}} {{\mathrm{max}}} \\ {E_i} \end{array}\mathop {\sum}\limits_{ < s_{\mathrm{a}},s_{\mathrm{b}} > \in R_i} F_i( < E_i(s_{\mathrm{a}}),E_i(s_{\mathrm{b}}) > ),$$

where *s*_a_, *s*_b_ can be words or concepts, *R*_*i*_ is a predefined relationship which bears some semantic and medical knowledge, *E*_*i*_ is a corresponding embedding space in which continuous vectors, learned by a proper function *F*_*i*_ (Supplementary Methods), are used to depict medical knowledge (examples see Fig. 6(a–c)). In this study, we totally used seven kinds of relationships based on the semi-structures of medical textbooks for multi-embeddings learning (Supplementary Methods).

In the “Guided Reading” phase we try to learn deeper and more subtle medical knowledge with supervised learning methods by using a large dataset of medical question-answers (MedQAs) as the training corpus. Given a medical question, we first get digest (a set of pieces of evidence related with the given question) through a “Fast Reading” process (Supplementary Methods and Supplementary Fig. 9) and then perform “Deep Reading”, supervised by the correct answer, over the digest and the given question. That is, $P(a_{{\mathrm{true}}}|Q,{\mathrm{evidence}}) > P(a_i|Q,{\mathrm{evidence}})$, where $a_i \in A_{{\mathrm{false}}}$ (a set of candidates but not including the correct answer). We learn a set of embeddings that capture reasoning knowledge on the MedQAs by the following equation

2 $$F_r(E_r(Q,{\mathrm{digest}},a_{{\mathrm{true}}})) > F_r(E_r(Q,{\mathrm{digest}},a_i)),$$

where *E*_*r*_ is a reasoning embedding space which maps the question *Q*, digest, and candidates into reasoning embeddings, and *F*_*r*_ is a function to measure the reasoning degree from question to candidates over digest. By supervised learning, we can capture deeper medical knowledge such as referring to disease from given complex clinical symptoms. Additionally, we enrich medical knowledge learned from “Free Reading” with delta-embedding learning *E*_*i*_ → *E*_*i*_ + *E*_Δ_. We learn a delta embedding *E*_Δ_ on top of a set of embedding *E*_*i*_ obtained by “Free Reading”, with structured regularization^22, 23^

3 $${\mathrm{loss}} = {\mathrm{loss}}_{{\mathrm{task}}} + c\left\| {E_{\mathrm{\Delta }}} \right\|_{21}$$

loss_task_ is a measure of the loss defined on the task (Here, the task is the medical question answering trained on MedQA). The embeddings are fine-tuned under supervision to give more precise representations. For example, the representation of medical term “nephritis” is turned into different representations which correspond to lupus nephritis, interstitial nephritis, glomerulus nephritis, allergic purpura nephritis, and pyelonephritis, respectively (Fig. 6d).

### Multi-layer reasoning

**Fig. 7:**
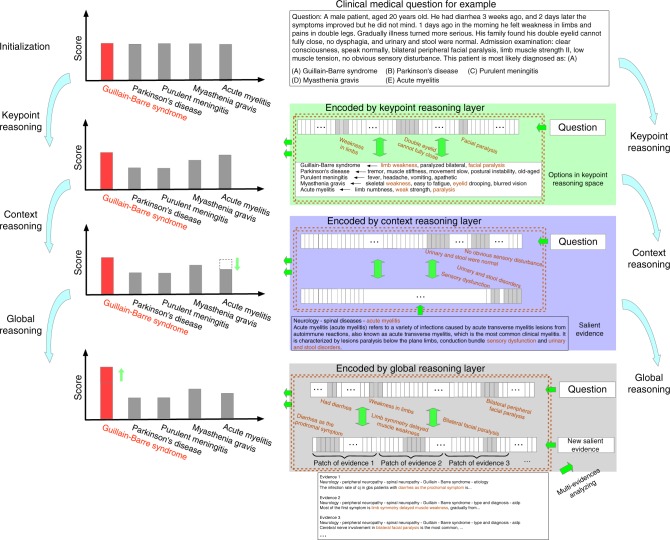
Example of multi-layer reasoning process. Take a clinical medical question for example, key points of the question are extracted and encoded by the keypoint reasoning layer to determine candidate answers A, D, and E; in the context reasoning layer, a salient evidence is used to exclude the incorrect answer E at sentence level; and in the global reasoning phase, more related pieces of evidence are analyzed to form a new salient one to support the true answer A

Here, we introduce a novel and robust multi-level reasoning cell based on neural networks. It reads questions and performs analysis and reasoning to answer the question using learned medical knowledge. The three level of reasoning are Keypoint Reasoning, Context Reasoning, and Global Reasoning. The layered architecture mimics a human’s reasoning and decision-making process. The lower level layers first utilize simple facts to perform direct and quick reasoning, then the latter layers take more information into consideration and perform reasoning that is more complex, indirect and obscure. By integrating reasoning from three layers we can produce a robust evaluation for all candidates of a given question and make a right choice (Fig. 7). Given a medical question, we first determine possible answers based on key points in the question.

4 $$Q \to \{ w_1,w_2,...,w_i\} \mathop{\longrightarrow}\limits^{{E_i}}a_{{\mathrm{true}}},$$

where {*w*_1_, *w*_2_…*w*_*i*_} are key points extracted from the question *Q*, and *E*_*i*_ are embeddings obtained from “Free Reading” and “Guided Reading”. In our Keypoint Reasoning network, we will not explicitly extract key points but assign them more importance with attentive strategies (Supplementary Methods and Supplementary Fig. 10). In Context Reasoning network we analyze the question using contexts and external knowledge from text (evidence from digest). The network reads medical text and extract a salient evidence from the digest which is the strongly relevant to answering the question:

5 $${\mathrm{digest}} \to s_{{\mathrm{salient}}}\mathop{\longrightarrow}\limits^{Q}a_{{\mathrm{true}}},$$

where *s*_salient_ is a salient evidence/sentence extracted from digest. We measure the degree by which the evidence supports the statement in the question, using attentive sentence modeling (Supplementary Methods and Supplementary Fig. 11). For some complex and difficult questions, there may be not any piece of salient evidence to directly support a true answer. We need to integrate a series of weak evidence in the digest to form a new strongly supporting evidence *s*_new_ to determine the true answer

6 $${\mathrm{digest}}\mathop{\longrightarrow}\limits^{{}}s_{{\mathrm{new}}}\mathop{\longrightarrow}\limits^{Q}a_{{\mathrm{true}}}$$

To realize this purpose, we introduce a global reasoning layer which examines all the documents in the digest, considers all pieces of possibly related information and makes a deep fusion to glue independent weak pieces of evidence into a strong one (Supplementary Methods and Supplementary Fig. 12). This reasoning layer is used in conjugate with the context reasoning layer, and the whole framework can be trained in an end-to-end fashion using gradient descent.

### Relation to WatsonQA

IBM Watson is a hallmark in open-domain question answering system (shortly called WatsonQA) with witnessed success. What makes it remarkable is its massively engineered architecture based on classical NLP pipeline^24–29^ and statistical approaches^30^. The sophisticated system involves hundreds of algorithms in a multi-staged fashion, which performs question analysis^31, 32^, candidate generation^33–35^, evidence gathering and analysis^36^, answer ranking^37^, and other engineering efforts^38–40^. The system extensively uses parsing^29^, semantic analysis^31, 32^, ranking algorithms^26, 37^, and feature engineering^24–29, 36^. Our proposed Med3R takes a different perspective which factors QA into two parts: knowledge representation learning and reasoning. Both parts are based on deep learning algorithms, and are learned end-to-end to fully exploit the representation power of deep neural networks and avoid the hassle of classical NLP pipelines.

### Code availability

Code used for this study is available from the corresponding author upon reasonable request. Exceptions are the medical knowledge representation learning source code and the global reasoning source code which are not publicly available an restricted by iFLYTEK Research. However, all experiments and implementation details are described in sufficient detail in the Methods and in the Supplementary materials.

## Electronic supplementary material

Supplementary Information

## Data Availability

The data that support the findings of this study are available from the corresponding author upon reasonable request. Exceptions are the medical corpus and question sets, which were used under license for the current study. These data are only available with permission from People’s Medical Publishing House (for medical corpus) and National Medical Examination Center (for question sets).
