# Supplementary material for: Master clinical medical knowledge at certificated-doctor-level with deep learning model
Source: Nat Commun. 2018 Oct 19;9:4352. doi: 10.1038/s41467-018-06799-6 (PMC6195515; doi:10.1038/s41467-018-06799-6)
Supplement: Supplementary file 1 — Supplementary Information [file 41467_2018_6799_MOESM1_ESM.pdf]

Supplementary Information for: ”Master clinical medical knowledge at certificated-doctor-level with deep learning model”

Ji Wu[1], Xien Liu[1], Xiao Zhang[1], Zhiyang He[2] & Ping Lv[3]

[1]*Department of Electronic Engineering, Tsinghua University, Beijing, China*

[2]*Medical Business Department, iFlytek Co.Ltd, Hefei, China*

[3]*Tsinghua-iFlytek Joint Laboratory, iFlytek Research, Beijing, China*

Supplementary Methods

Data set. The large-scale medical corpus consists of text materials on medical subjects from multiple sources. A major portion of the corpus is prepared from various publications. A total of 32 published books are used, including textbooks for medical school students, reference books for medical practitioners, and guidebooks. All text materials is extracted from these books and is divided by paragraph. The content structure of the book is parsed, and nested levels of chapter titles are extracted. This title information is appended to corresponding text paragraphs as metadata. Each paragraph along with its metadata is stored as a "document". A total of 243,712 documents is extracted from these publications. The **MedQAs dataset** is a collection of exam problems with ground-truth answers. A total number of over 270,000 problems are collected from the internet and from published materials such as exercise books. The problems collected are not official test problems from past examinations, but could be related to those. We filtered out any incomplete or duplicate problems. Questions in these problems have an average length of 27.4 words. Each problem has exactly 5 candidate answers. A training/valid split is created for supervised training of models. The problems in the valid set are chosen based on an estimated fidelity of appearance in past exams. A small subset of problems was chosen based on their source and context that indicate their possible appearance in past exams. The valid set is constructed to approximate performance in real exams. The final training and valid set contains 222,323 and 6,446 problems respectively.

Similarity degrees. We calculate the similarity degrees of NMLEC 2017 questions against our training dataset with following manners. For each question from NMLEC 2017, we computes its

Levenshtein ratio against all questions from the training set of MedQA, then averaged the top five values as its similarity degree. A lower value means more dissimilarity.

Embedding spaces. The word2vec algorithm treats text materials as being unstructured and only depicts general neighboring relationship between words, thus embeddings learned by word2vec mainly describe general language and commonsense knowledge. We found that text materials in medical domain are not completely unstructured but are often semi-structured. For example, words in medical textbooks are organized into different semantic units, such as chapter, section, subsection, paragraph, and sentence etc. Therefore we can define rules to organize text from these medical corpus into separate text fragments with relationships based on the semi-structured information. Besides neighboring relationship (R_1 used by word2vec), in this study we also used another three relationships to describe long range contextual dependency. They are 1) R_2: two words in the same sentence; 2) R_3: two words in the same paragraph, 3) R_4: two words in the same section. Since diseases play important roles in understanding medical knowledge. We extracted about 1174 diseases and their content description from medical textbooks involving more than ten subjects, such as infectious diseases & STD, pediatric, internal medicine, stomatology, respiratory medicine, chirurgery, obstetrics and gynecology, dermatovenerology, neurology, and otorhinolaryngology head and neck surgery etc. In textbooks, each disease (usually as a subtitle) is followed by a paragraph (content description) which comprehensively describes the characteristics of the disease. We split the paragraph into several sub-paragraphs which describe the disease at views of ”symptom”, ”examination”, and ”differential diagnosis”, respectively. The ”symptom” sub-paragraph mainly describes symptoms of the disease; the ”examination” sub-paragraph mainly

describes which examinations are needed to make a definite diagnosis for the disease; and the "differential diagnosis" sub-paragraph presents content of how to differentiate the disease from other similar diseases. Based on these, we further defined another three relationships: 1) R_5: disease and its "symptom" description; 2) R_6: disease and its "examination" description; 3) and R_7: disease and its "differential diagnosis" description. All of our used relationships for multi-embedding learning are summarized in the the Supplementary Table 2.

Embedding of words and concepts. In general applications, it's enough to build embeddings on words or characters. However, in medical domain concepts are so important for presenting medical knowledge that we need build embeddings on concepts. To achieve this goal, we firstly employed NER technology to annotate medical concepts (such as diseases, symptoms etc.) for textbook materials. For annotated medical concepts we build embeddings for them, and for other non-concept words, we still build embeddings on words. That is, concept embeddings and word embeddings are learned simultaneously according the relationships defined in Supplementary Table 2.

Embedding learning. According to equation (1) presented in the Methods section of main text, given two words/concepts (or one is a word and the other one is a concept) s_a, s_b, and $< s_\mathrm{a}, s_\mathrm{b} >\in R_i$ we obtain their embeddings by maximizing their conditional probability $P(s_\mathrm{a}|s_\mathrm{b})$ with a proper function F_i. For simplicity, in this study F_i is defined with a softmax function:

$$P(s_\mathrm{b}|s_\mathrm{a}) = F_i(< E_i(s_\mathrm{a}), E_i(s_\mathrm{b}) >) = \frac{e^{(E_i(s_\mathrm{a}))^T (E_i(s_\mathrm{b}))}}{\sum_{s_\mathrm{w} \in V} e^{(E_i(s_\mathrm{a}))^T (E_i(s_\mathrm{w}))}}, \tag{1}$$

where $E_i(s_\mathrm{a}), E_i(s_\mathrm{b})$ are embeddings of s_a and s_b, respectively. $(E_i(s_\mathrm{a}))^T$ is transpose of $E_i(s_\mathrm{a})$.

Embedding combining. Multiple embeddings are combined by concatenation when used as input to neural network components in out model. Although there exists more sophisticated ways of combining embeddings, we found in our experiments that a simple concatenation here is robust enough in achieving desired performance.

NMLEC. Medical licensing examination is a feasible and appropriate manner, officially adopted by many countries over the world to assess a physician's mastering medical knowledge and the ability to apply the knowledge, and the principles to solve medical problems. In China, NMLEC is annually organized by NMEC to assess doctors' quality. In China, every year more than 530,000 candidates participate into the exam. The General Written Test (GWT) of NMLEC is produced by board-certified and experienced medical experts who are employed by NMEC. GWT consists of 600 choice-questions with four categories A1, B1, A2, and A3/A4 (details in Supplementary Figure 2). All of the questions mainly cover medical knowledge from preclinical medicine, medical humanities, preventive medicine and clinical medicine (details in Supplementary Figure 1).

Fast reading: context-based vector space text retrieval. The quality and the relevance of the retrieved digest is an important factor determining the performance of the reasoning module. The aim of the retrieval system is to extract semantically relevant information from the large medical corpus. Traditional text retrieval methods such as BM25 lacks the ability to distinguish relevancy at the semantic level. We developed a vector space retrieval model based on context vectors. Context vectors are representations of words in context, which captures the syntactic structure, e.g. phrases, and semantic representation, e.g a specific word sense. Context vectors $\mathbf{c}_i$ are generated from word

embeddings $\mathbf{w}_i$ using a bidirectional LSTM network, which is trained to model context in the reasoning module. Retrieval of related documents from the text corpus is performed in pure vector space. This consists of a two-step process. In the first step, context representations $\mathbf{c}$ are generated for all documents in the text corpus: each document is first embedded into vector space using one set of word embeddings, then processed by a bidirectional-LSTM network. Word embedding is learned in the free reading module, unsupervisedly on all text corpus using skip-gram and has dimension 200. LSTM network is extracted from the context reasoning module after supervised training on the QA dataset. The parameters of the 256-dimensional bidirectional-LSTM network used in the input layer of the context reasoning module is extracted. We use this LSTM network as a universal context modeling network to process all text materials:

$$[\mathbf{c}_0, \mathbf{c}_1, ..., \mathbf{c}_S] = \overleftrightarrow{\text{LSTM}}[\mathbf{w}_0, \mathbf{w}_1, ..., \mathbf{w}_S] \tag{2}$$

The output vector sequence of the LSTM network is associated with the original text document and is collected in a vector database V. The total number of vectors in V is 22,740,576, equal to the total number of words in the text corpus. An index based on IVFADC is constructed using k-means to cluster all vectors into 4096 cells. Product quantization is used to compress each vector to 64 bits. Indexing of vectors and searching are performed with faiss toolkit. In the next step, given a question q as query, first the context vectors $\mathbf{c}_i$ of the query are generated using the same LSTM network as above. For each context vector, $N = 2000$ nearest neighboring vectors (by Euclidian distance) are retrieved from V. The retrieved vectors are grouped by the original document they belong to, and the documents are scored by relevancy. The relevancy score of a document d w.r.t.

a query q is defined as:

$$s(q,d) = \frac{1}{L_q}\sum_{i=0}^{L_q}\delta_i(\frac{1}{D_i+\alpha}+\beta) \tag{3}$$

where δ_i is the indicator of a match found at position i of query (L_q being the length of query in words):

$$\delta_i = \begin{cases} 1, & \text{if there is a vector } \mathbf{v} \text{ within the } N \text{ nearest neighbors of } \mathbf{c}_i, \\ & \text{and } \mathbf{v} \text{ is a context vector of } d \\ 0, & \text{otherwise} \end{cases} \tag{4}$$

and D_i is the Euclidian distance between $\mathbf{c}_i$ and the matched context vector $\mathbf{v}$. Parameters α and β are empirically set to 1.0 and 2.5 respectively. The scoring function is meant to simply average the degree of matching of each word in the query to the document. The distance between two context vectors reflect the semantic similarity of two words and their surrounding context. Measuring context distances instead of word co-occurances alleviates the conditional independence assumption of words in traditional retrieval methods and makes semantically related documents rank higher. The top 5 documents that rank highest from each source of text is returned as a digest of related information in the text corpus given the question as a query.

Keypoint reasoning. The keypoint reasoning module (Supplementary Figure 10) is designed to use key points in a question to infer its correct answer. One of the main challenges is to recognize and extract key points from the question. Here, we do not explicitly extract key points but adapt an implicit manner that important words (playing key roles in inferring answer) are given high weight values and less important words are assigned lower weight values. To realize this purpose, we employ an attention learning mechanism which is a fashion strategy commonly used in deep

learning models recently. Firstly, we assume that every words are useful and establish a reasoning relationship between question and its answer at word level with a Reasoning Matrix (RM) $M \in R^{m \times n}(m, n$ are the numbers of answer's words and question's words):

$$M(i,j) = (v_i^o)^T (v_j^q), \tag{5}$$

where $v_i^o \in R^{D \times 1}$ is an unit embedding/vector ($||v_i^o||_2 = 1$) of the ith words in the answer, and $v_j^q \in R^{D \times 1}$ is an unit embedding ($||v_j^q||_2 = 1$) of the jth words in question. That is, the above equation calculates reasoning degree between words from question and words from answer by using cosine distance. For simplicity, we denote M_1, M_2, and M_3 is the corresponding RM from the first layer, the second layer, and the third layer, respectively. Then, we will introduce two kinds of attention-based filter strategies to reduce noisy/undesirable words and pay more attention on key-point words (which are important to infer correct answer). We notice that there exists a kind of noisy words which disturb making correctly reasoning. We shortly called it ”synonymy-noise” which describes a phenomenon that there exist the same words or synonymy words both in question and answer. However, here we want to establish a reasoning relationship between words from question and words from answer, such as words describing illness Symptoms in question and words depict corresponding treatment in answer. The synonymy relationship between words from question and words from answer is not expected. According to equation (5), word pairs having synonymy relationship (or they are the same word) have a large value. To reduce ”synonymy-noise”, we introduce an Internal-Attention (I-A) strategy to produce an Attention Matrix (AM) A_1 based the first layer reasoning matrix M_1

$$A_1 = \text{sigmoid}(k_1(M_1 + \tau_1)) \cdot \text{sigmoid}(-k_1(M_1 - \tau_1), \tag{6}$$

where k_1 is a scale value, $\tau_1 \in (0,1)$ is a threshold parameter to determine which degree in A_1 to be reduced. Since we want to reduce high degree (always generated with "synonymy-noisy" words), τ_1 promotes to be a large value. In this study, empirically $k_1 = 10$, and $\tau_1 = 0.9$. It must be pointed that according to our experiments k_1 can be any value of the range $(5, 30)$, and τ_1 can be value ranging in $(0.75, 0.95)$. The model performance performance decline can be omitted compared to the best setting model. After obtaining the attention matrix A_1, we transfer the "prior knowledge" obtained from the first layer into the second layer by using a simple External-Attention (E-A) strategy

$$M_2 = M_2 \cdot A_1 \tag{7}$$

Besides "synonymy-noisy", we also notice that many words in question have little reasoning relationship to answer, such as stop words which only perform an grammatical function in the sentence. We shortly called this kind of noise words "irrelevance noise". We assume that if two words (one is from question, the other is from answer) have no/little reasoning relationship, their reasoning degree (calculated by the second layer reasoning matrix A_2) is a little value. Therefore, we reduce the "irrelevance noise" by an I-A strategy in layer two

$$A_2 = \mathrm{sigmoid}(k_2(M_2 - \tau_2)) + \mathrm{sigmoid}(-k_2(M_2 + \tau_2), \tag{8}$$

where k_2 is a scale value, and τ_2 is a threshold parameter to determin which degree to be reduced. In this study, empirically $k_2 = 20$, $\tau_2 = 0.05$. According to our experiments k_1 can be any value of the range $(10, 30)$, and τ_1 can be value ranging in $(0.01, 0.1)$. we also transfer the "prior knowledge" obtained both from the second layer and the first layer into the third layer by using the

E-A strategy

$$M_3 = M_3 \cdot A_2 \cdot A_1 \tag{9}$$

To further enhance the effectiveness of reducing ”irrelevance noise”, we employ the same calculation (8) used in the second layer to obtain the attention matrix A_3. Ultimately, we employ an ensemble manner to calculate the Keypoint Matrix (KM) K

$$K = A_3 \cdot (M_1 + M_2 + M_3) \tag{10}$$

KM represents reasoning degree at word level, which gives large degree value to key point words and little value to noisy words. We obtain the total reasoning degree value r between question and answer by

$$r = \sum_i \sum_j K(i, j) \tag{11}$$

Context reasoning. The purpose of context reasoning module is to determine the salient evidence from the digest and use that salient evidence to reason about the answer. The module is an end-to-end neural network inspired by recent advances in machine reading comprehension. The model takes question text and digest documents as input, performs analysis on individual document basis and finally gives an output representing answer probabilities. The context reasoning model is itself a multi-layer neural network. The encoding layer, the first layer of the network consists of a word embedding lookup and a bi-directional LSTM network. Input text is a question-answer pair Q and a set of digest documents $\{D_0, D_1, ..., D_N\}$. The question and answer text is simply concatenated together. A set of embeddings E_i generated in the Free Reading and Guided Reading module is used in word embedding lookup. The embedded text are concatenated along the feature dimen-

sion and given as input to a 256-dimensional bi-directional LSTM network. The LSTM network encodes the text to give contexual text representations. We analyzed the properties of these representations from trained model, and found that they give fine-grained semantic information such as differentiated word senses and phrase structures. LSTM networks with the same parameters are used to process the question-answer pair and the documents to facilitate parameter sharing in the network. The second layer of the network is mainly attention network used to find and extract relevant information from documents. To model the semantic correspondence between question Q and documents D_n, a pairwise matching matrix M (for each document separately) is calculated as the dot-product of the context embeddings between Q and D_n:

$$M_n(i,j) = Q(i) \cdot D_n(j) \tag{12}$$

A dual-path attention structure is used to match information between question and documents and extract relevant information given the matching matrix M. Attention is first performed column-wise, where each word $Q(i)$ in the question gets a summarization read $R_n^Q(i)$ of related information in the document D_n:

$$\alpha_n(i,j) = \text{softmax}(M_n(i,1), ..., M_n(i,L_D))(j) \tag{13}$$

$$R_n^Q(i) = \sum_{j=1}^{L_D} \alpha_n(i,j) D_n(j) \tag{14}$$

Attention is also performed row-wise to summarize relevant information in the reversed direction, from question to document:

$$\beta_n(i,j) = \text{softmax}(M_n(1,j), ..., M_n(L_Q,j))(i) \tag{15}$$

$$R_n^D(j) = \sum_{i=1}^{L_Q} \beta_n(i,j)Q(i) \tag{16}$$

Besides attention reads, a set of matching features is used, which is extracted directly from the matching matrix M with a two layer convolutional neural network. The CNN network has 3x3 kernel and 2x dilated convolution in the second layer. The motivation of the feature extractor is to make use of values and patterns in matching matrix M, which contain information about matching of local text regions, such as different phrasing of a same fact. The next layer is a pair of bi-directional LSTM network of 256 units with per-word input gating. This layer takes all output from the previous layer as input: the concatenation of context vectors of question and each document, the summary reads of question and each document, and matching features. The per-word gating identifies important words in question and documents, and the LSTM network reads over question and extracted information (attention reads) from each document, performs reasoning in semantic level to determine the support of each document to the question. Each document is processed separately, and for each document the output of the LSTM network is pooled along the sequence with mean and max pooling to produce a summary vector representing the overall supporting relation between the question and the document. The final component of the Context Reasoning module is a decision network. First the most salient evidence is decided using a gating layer. The support vector of the selected evidence is given to a multi-layer feed-forward network, which evaluates the correctness of the candidate answer given support from the salient evidence. Correctness of the answer is represented by a scalar output given by the MLP network. The whole Context Reasoning module is trained end-to-end on the training set of medical QA dataset, using

the groundtruth answer as supervision. Note that we don't have labels for salient evidences, and the model's ability to select salient evidences naturally emerge when trained on the end task. To achieve this we first let the gating layer weight the different documents and produce a weighted mix of evidences for the decision network, then train the model to some point and replace the weight mix with the single most weighted document (the identified salient document). The model is implemented in Tensorflow with LSTM network implementation from nvidia's CUDNN library. Training used Adam optimizer and dropout of rate 0.2 between layers. Hyperparameters are chosen using the validation set. Training takes roughly 18 hours on a single GTX 1080 GPU.

Global reasoning. A graph-based approach is employed to effectively distill evidence from documents. An explicit graph is constructed from question, answer and all documents from the digest to model the relation between evidence words. Words form the nodes of the graph, and an edge denotes the co-occurrences of a pair of words within a document. Each node is given a weight W^{N} equal to the inverse document frequency of the word. A weight W^{E} is also given to each edge. Co-occurrences within a shorter window is given a higher weight. For co-occurrences within multiple documents, the edge weight is given as

$$W^{\mathrm{E}} = (W^{\mathrm{N}}_{\mathrm{left}} + W^{\mathrm{N}}_{\mathrm{right}}) \sum_i W^{\mathrm{E}}_i W^{\mathrm{D}}_i \tag{17}$$

where W^{E}_i is the weight of the co-occurrences of word *left* and in *right* the i-th evidence, and W^{D}_i is a weight given to the evidence (to penalize longer evidences). The maximum spanning tree (MST) containing all nodes representing words in question an answer is generated from the graph. The nodes in the graph are words directly or indirectly related to the question, and form a new evidence that supports the question. The weights in the MST and are used to evaluate the

supporting strength of the evidence to the question-answer pair. An implicit graph is also generated to calculate relevance in embedding space, which can be regarded as the continuous version of the graph-based approach. Matching matrix are calculated for each question-evidence pair and each evidence-evidence pair:

$$M_n^{\mathrm{Q}}(i,j) = Q(i) \cdot D_n(j) \tag{18}$$

$$M_{mn}^{\mathrm{D}}(i,j) = D_m(j) \cdot D_n(j) \tag{19}$$

These matrix are aligned along occurrences of words and are pooled together to summarize relevant pieces from all evidences. A evidence synthesis network combines relevant pieces of information from several evidences into one single evidence. The pieces are iteratively selected to construct a new evidence E_{new}:

$$E_{\mathrm{new}} = [\mathbf{e}^0_{a^0_0}, \mathbf{e}^0_{a^0_1}, ..., \mathbf{e}^1_{a^1_0}, \mathbf{e}^1_{a^1_1}, ...] \tag{20}$$

where $\mathbf{e}^n_{a^n_i}$ represents the a^n_i-th vector in the n-th evidence, and is found by maximizing question-evidence relevance (values in M_n^{Q}) and minimizing evidence-evidence overlap (values in M_{mn}^{D}). The new evidence is then matched with the question representations to generate a supporting matrix, which integrates support from all evidences. Reasoning reuses part of trained network from context reasoning module. The final output vector represent the overall support of the evidences to the statement in question.

Baseline methods. We adapted or developed several baseline methods for experiments, including: 1) **R-net**: A reading comprehension model achieving previous state-of-the-art single model result

on the SQuAD dataset[1]. It has an architecture that stacks a question-to-document attention layer and a document-to-document attention layer. Because the model is originally used on SQuAD, which has a single input document, we concatenated the digest documents as input to the model. The final prediction layer is also replaced with a pooling layer to generate a scalar score.

2) **Neural reasoner**: A framework for reasoning over natural language sentences, using a deep stacked architecture. It can extract complex relations from multiple facts to answer questions. We supply individual documents in the digest to the model as different evidences.

3) **Iterative attention**: This model has a universal architecture that does not limit its use to a specific task. It uses attention to read from question and document alternatively, and the read is performed iteratively then the final state is used to make prediction. The model is directly applicable to our Medical QA task.

The above three models are all end-to-end neural network models and are trained using the same Medical QA dataset as our own model. They share the unsupervised word embeddings in "Free Reading" of Med3R framework.

A WatsonQA system. the WatsonQA system is a pipelined system based on evidence retrieval and analysis. It has a hand-crafted retrieval system based on Lucene to retrieval potential evidences from the corpus. The evidence analysis is based on word co-occurence graph between question and a document. The graphs are merged into a single graph to represent relation between question and multiple evidences. The nodes and edges of the graph contain multiple types of features as weights. Finally a set of graph feature extractor is used to summarize the evidence graph into a vector for

[1]https://rajpurkar.github.io/SQuAD-explorer/

scoring the candidate answers.

Model training. The unsupervised embeddings in the ”Free Reading” module are first trained on the medical corpus. Next, the multi-layer reasoning module is trained on the Medical QA dataset in an end-to-end fashion. The three components of the reasoning module is individually trained, using the correct answer as supervision. At this stage the vector space retrieval model in guided reading module is bypassed and a BM25 based text retrieval model is used instead to provide a document digest. For the ”Guided Reading” module, the vector space retrieval model is constructed by transferring the parameters of the LSTM network from the trained context reasoning model. The supervised embedding learning in deep reading contains 2 components: the reasoning embedding is trained together with the keypoint reasoning module to learn embeddings for keywords, and the supervised-refining of embedding is done during training of context reasoning module. The whole framework is thus largely trained end-to-end except for the ”bootstrapping” of the vector space retrieval.

Supplementary Figures

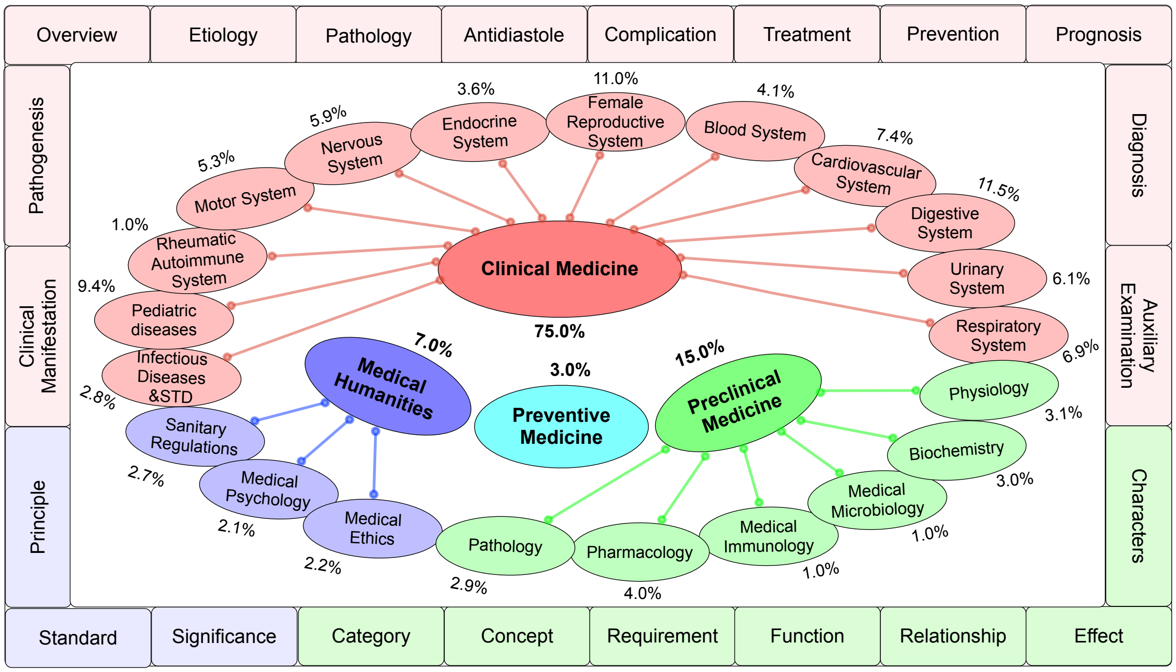

Supplementary Figure 1 | **The written test of NMLEC.** The written test of NMLEC consists of 600 multiple-choice medical questions (600 points, one point per question), covering medical knowledge from four parts: synthesis of clinical medicine (75%), synthesis of preclinical medicine (15%), synthesis of medical humanities (7%), and synthesis of preventive medicine (3%). The four parts cover more than 20 medical subjects, and are examined by more than 20 examination points such as etiology, antidiastole, prognosis, diagnosis etc.

Category	Description	Ratio
A1	**Single statement best choice**: single statement questions, 1 best answer, 4 incorrect or partially correct answers	36.8%
B1	**Best compatible choice**: similar to A1, with a group of candidate answers shared in multiple questions	11.2%
A2	**Case summary best choice**: questions accompanied by a brief summary of patient's medical record, 1 best choice among 5 candidate answers	35.3%
A3/A4	**Case group best choice**: similar to A2, with information shared among multiple questions	16.7%

(A1) **Question**: The pathology in the pancreas of patients with type 1 diabetes is:
a. Islet cell hyperplasia
b. Islet cell necrosis
c. Interstitial calcification
d. Interstitial fibrosis
e. Islet cell vacuolar degeneration
(A2) **Question**: Male, 24 years old. Frontal edema, hematuria with cough, sputum with blood for 1 week. bp 160/100 mmhg, urinary protein (++), rbc 30/ hp, ... (omitted text) Ultrasound show kidney size increase. At present the most critical treatment is:
a. Hemodialysis
b. Prednisone
c. Plasma exchange
d. Gamma globulin
e. Prednisone combined with cyclophosphamide
(A1) **Question**: The preferred treatment for pregnant women with genital Chlamydia trachomatis infections is:
a. Doxycycline
b. Ceftriaxone
c. Ofloxacin
d. Erythromycin
e. Penicillin
(A2) **Question**: Male, 46 years old. Sudden knife-like pain in upper abdomen 4 hours after a full meal. The pain soon spread to the whole abdomen and was persistent. Physical examination: abdominal muscle tension, abdominal tenderness, rebound pain positive, liver dullness narrowing, shifting dullness positive, bowel sounds disappear. Blood test showed an increased WBC and neutrophils. The most likely diagnosis is:
a. Acute hemorrhagic necrotizing pancreatitis with infection
b. Acute volvulus
c. Acute gallbladder perforation with localized peritonitis
d. Perforated ulcer with diffuse peritonitis
e. Perforated appendix with localized peritonitis

Supplementary Figure 2 | Examples of the written test of NMLEC and it's categories. The written test of NMLEC totally has 600 questions, consisting of four categories A1, B1, A2, A3/A4.

“智医助理”测试结果反馈

为促进我国人工智能在医疗卫生领域中的研发和应用，国家卫生计生委和安徽省政府支持科大讯飞股份有限公司（以下简称“科大讯飞”）研发面向基层医生的临床决策支持系统（以下简称“智医助理”），国家医学考试中心（以下简称“医考中心”）在 2017 年医师资格考试医学综合考试期间，运用临床类别执业医师医学综合考试试卷对“智医助理”应答能力进行了测试。

现将测试结果反馈如下：

一、测试过程

2017 年 8 月 26 至 27 日，在全国医师资格考试期间，医考中心在其办公楼内设置测试专用考场，顺利完成了“智医助理”测试工作。

为确保测试顺利，安徽省卫计委、科大讯飞和医考中心签订三方协议，成立了专门工作小组，多次召开专题会议，研究测试流程、突发情况处置、保密要求和公证流程等事宜。考前还组织了模拟演练，用模拟试卷对“智医助理”测试设备、考务管理和测试流程进行模拟演练。

国家医学考试中心为本次测试单独设置了考场，考场内的安全保密措施严格，开启了通信信号屏蔽仪，测试设备严禁与互联网连接、严禁具有无线上网功能，所有参与测试的工作人员均签署了《保密承诺书》，安检后方进入考场。

测试时间与正式考试基本同步。测试过程严格按照医师资格考试医学综合考试考务工作规程的要求完成考场监考、试题交接、答卷评分等工作，并由北京市国信公证处全程公证。

测试期间，医政医管局张宗久局长和安徽省卫生计生委于德志主任亲临测试现场指导工作。

二、测试结果

（一）测试对象

“智医助理”。

（二）测试工具

2017 年临床执业医师资格医学综合考试试卷。

（三）测试内容和题量

测试内容由基础医学、临床医学、医学人文、预防医学四个模块组成。测试总题量 600 题，分为四个单元，每个单元 150 题，全部为单选题。

（四）测试成绩

“智医助理”医学综合考试的总成绩为 456 分，高于全国各类考生的平均分。2017 年全国考生最高分为 533 分。

（五）初步印象

1. “智医助理”医学综合考试测试成绩位居各类考生前列。
2. “智医助理”在临床医学模块表现最好。
3. “智医助理”对记忆型试题的掌握率最高。
4. “智医助理”在病例型试题上的表现略低于非病例型试题，对需运用多个知识点答题的试题掌握率相对较低。

Test Result of “Intelligent Doctor Assistant”

In order to promote the research and application of artificial intelligence in the field of medical treatment and public health in our country, NHFPC (National Health and Family Planning Commission) and the government of Anhui province support IFLYTEK CO., LTD in researching and developing intelligent clinical decision support system (Intelligent Doctor Assistant) for PCPs (Primary Care Physicians). In the China National Medical Licensing Examination (NMLE) of year 2017, National Medical Examination Center (NMEC) tested the performance of Intelligent Doctor Assistant using the examination for clinical physician.

Below are the feedbacks:

1. Procedure of the Tests

From 26th to 27th of August, during the National Medical Licensing Examination of year 2017, the National Medical Examination Center set up a test room in its office building and successfully finished the test of Intelligent Doctor Assistant. To ensure the test to run smoothly, Anhui Provincial Health and Family Planning Commission, IFLYTEK CO. together with the NMEC signed up a tripartite arrangement and established a special working group. In addition, several special meetings were held to investigate test procedures, emergency management, confidentiality requirements and notarization procedures. Before the test, the simulation exercises were organized, and the test equipment, test management and test procedures were simulated with simulated test paper. NMEC set up a separate examination for the test with security and confidentiality enforcements. Wireless communication was prohibited with the use of signal jamming facilities. Absolutely no internet access was granted for the test system. All staff involved in testing signed a Confidentiality Commitment, and are security checked before entering the test room.

The test time was in sync with the official exam. Test process was in strict accordance with the NMLE work procedures including exam invigilation, handover of examination questions, paper scoring etc. And the procedure was notarized by Beijing Guoxin Notary Public Office. During the test, the director of the Medical Administration Zhang Zongjiu and the director of the Anhui Provincial Health and Family Planning Commission visited the site to guide the test.

2. Result of the Test

2.1 Test Object

Intelligent Doctor Assistant

2.2 Testing Tool

National Medical Licensing Examination for clinical physician, 2017

2.3 Test Content and Volume

The test consists of four modules: basic medicine, clinical medicine, medical humanities and preventive medicine. The total number of problems in the test is 600. It is divided into four units. Each unit has 150 problems.

2.4 Test Performance

The total score of NMLE of the Intelligent Doctor Assistant was 456, higher than the average score of all kinds of candidates in the country. In 2017, there highest score of candidates is 533 in the country.

2.5 Initial Impression

(1) The NMLE results of Intelligent Doctor Assistant ranks among the top of all kinds of candidates.
(2) Intelligent Doctor Assistant performs best in clinical medicine module.
(3) Intelligent Doctor Assistant has the highest performance in memorization test.
(4) In the case type test, Intelligent Doctor Assistant’s performance is slightly lower than that of the non-case test. The performance is lower for the test questions that require the application of multiple key points of knowledge.

(a) Summary of test results (Chinese) **(b)** Summary of test results (translated into English)

Supplementary Figure 3 | Summary report of our Med3R AI-system (“智医助理” in Chinese, “Intelligent Doctor Assistant” in English) participating into the written test of NMLEC 2017, officially offered by NMEC.

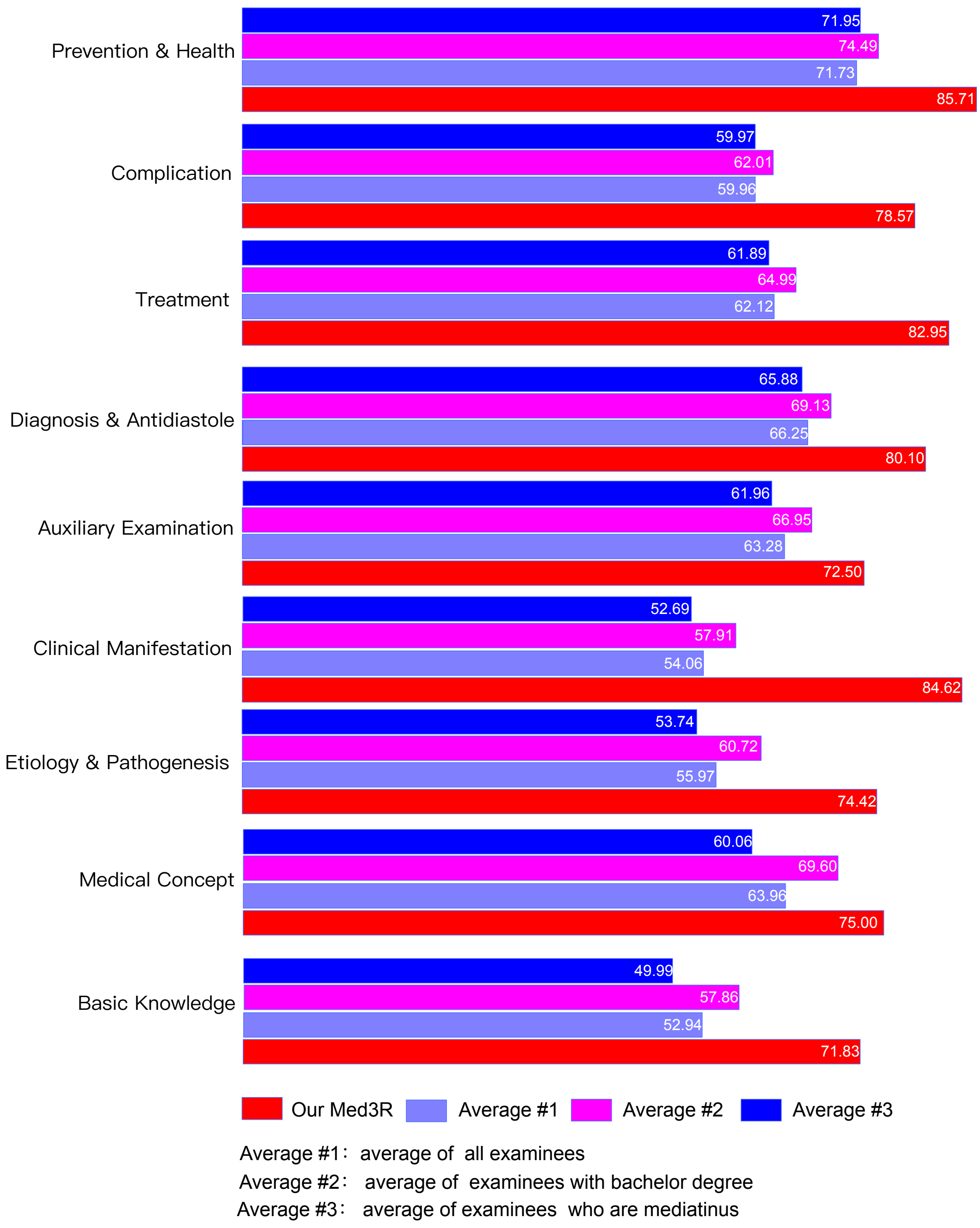

Supplementary Figure 4 | Comparing analysis to the synthesis of clinical medicine of NMLEC 2017. Detail comparison analysis is conducted over the 9 categories (including “Prevention & Health” , “Complication”, “Treatment” etc.) of the synthesis of clinical medicine to illustrate the superior performance our Med3R model.

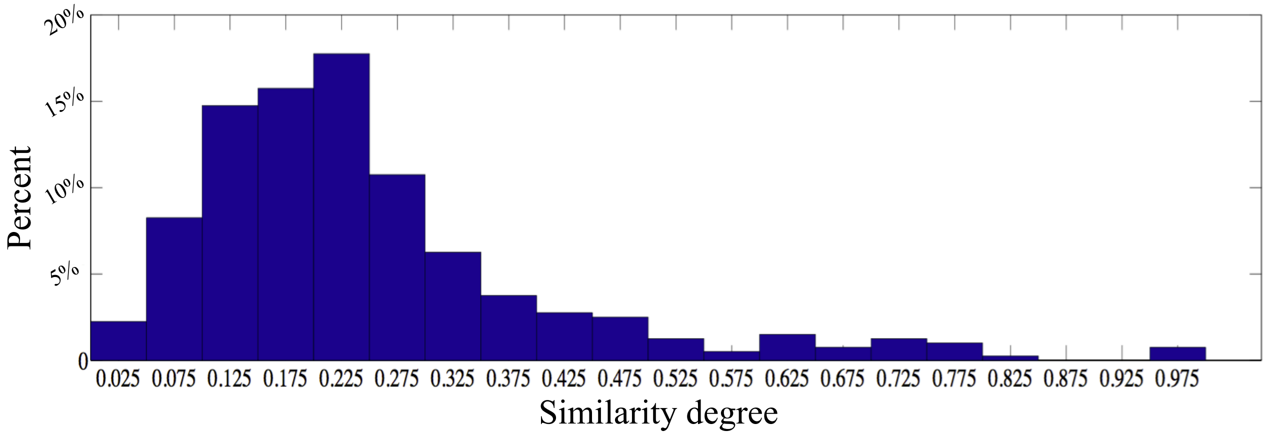

Supplementary Figure 5 | Question similarity between NMLEC 2017 and MedQAs. The degrees of similarity between questions from NMLEC 2017 and questions from the training set of MedQA are measured with Levenshtein distance, where a lower value means more dissimilarity. The distribution of the similarity degrees is presented here.

EMR data example (in Chinese)

主诉: 咽喉疼痛5天

现病史: 患者于5天前着凉后咽喉疼痛，吞咽时加重，未出现鼻塞、声音嘶哑、咳嗽、呼吸困难、发热症状，自起病以来饮食睡眠良好。

疾病码: J06.9

EMR data example (translated in English)

Chief complaint: Sore throat for 5 days.

History of present illness: After catching a chill 5 days ago, the patient began to suffer from sore throat, which worsened when swallowing. No nasal obstruction. No hoarseness. No cough. No fever. No expiratory dyspnea. The patient has no difficulty in somnus and eating.

Disease code: J06.9

Supplementary Figure 6 | Example of EMR data sample. EMR data are collected from outpatient services of several areas (where qualified doctors are shortage) in China. An EMR sample is mainly consist of two parts: 1) Chief complaint (very brief description of the patient's illness symptom) and 2) History of present illness (comprehensive description of the patient's illness).

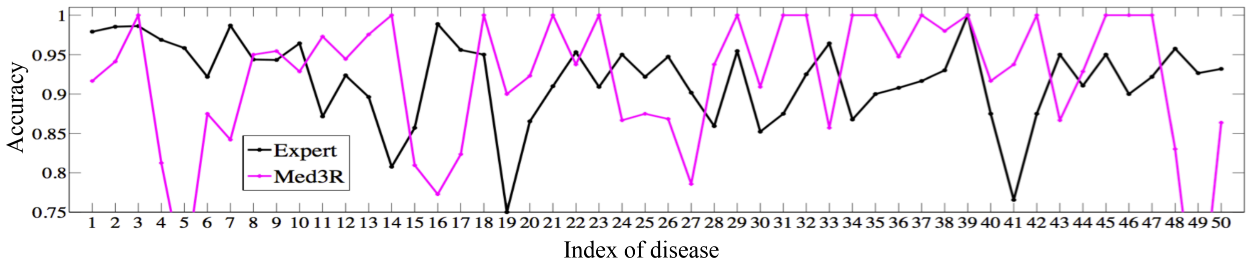

Supplementary Figure 7 | Accuracy comparison between our system Med3R and medical experts over 50 diseases. Here Med3R NMLEC system is directly used for diagnosis (without fine-tuning on EMR data). The accuracy of expert listed here is the average accuracy of four medical experts (Details about four medical experts annotation accuracy is given in Supplementary Figure 8). The total accuracy of Med3R (92.04%) is almost comparable to experts' performance (92.15%). When adapted on EMR data, the performance of Med3R is more superior than experts' accuracy (Details see Figures 3&4).

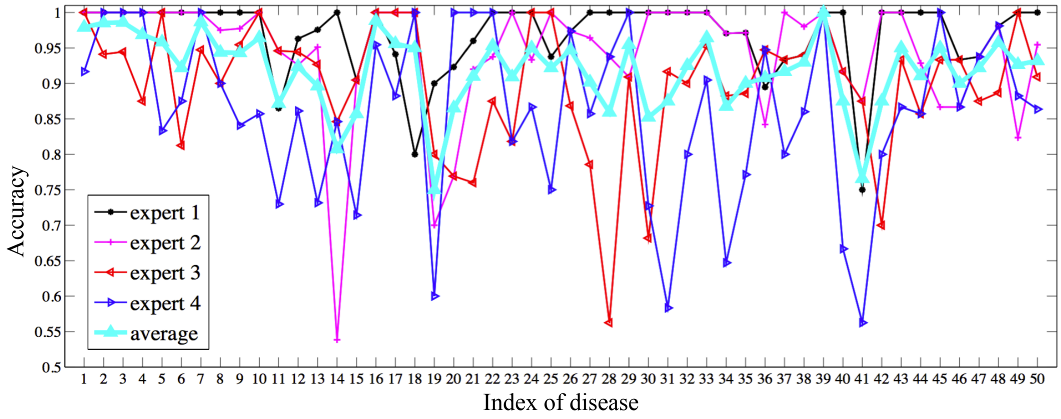

Supplementary Figure 8 | Annotation accuracy of four medial experts over 50 diseases. More details about the four medical experts are presented in Supplementary Table 1.

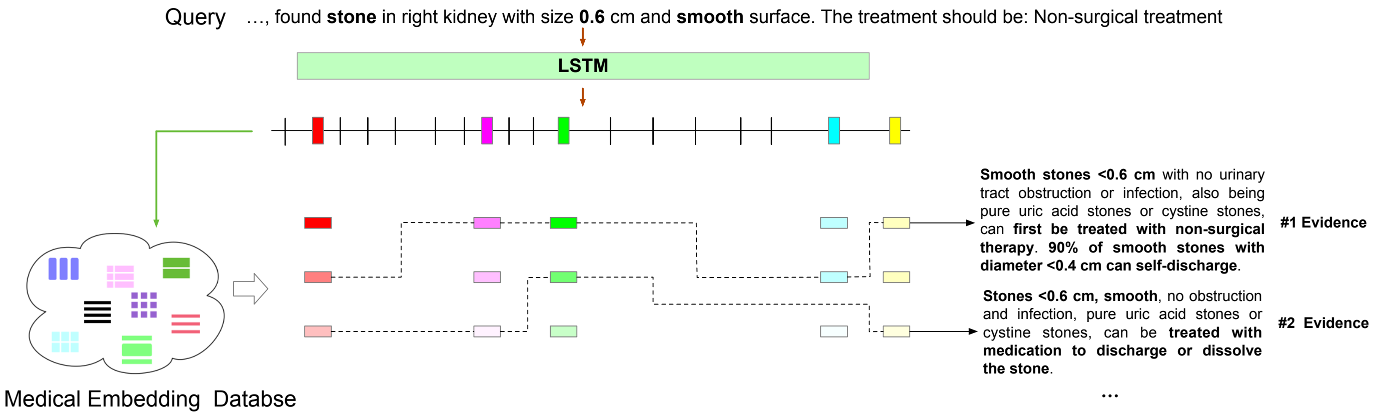

Supplementary Figure 9 | Fast reading: context-based vector space text retrieval. The query and all the documents are feed into an LSTM network to generate context vectors. A context vector represents a word in its context. Nearest neighbor retrieval is performed in vector space to find semantically similar contexts, and relevant contexts from the same document is grouped together. A simple scoring function gives the measure of relevancy and ranks the retrieved documents.

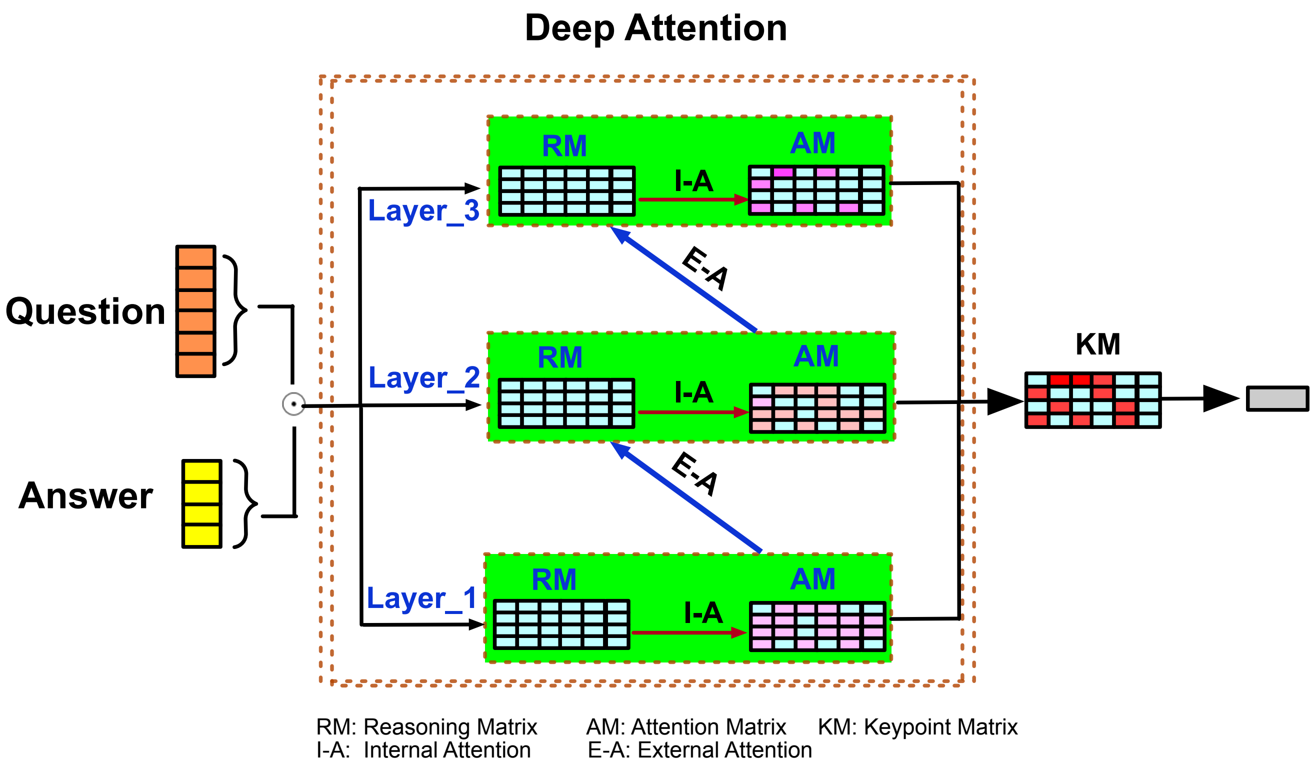

Supplementary Figure 10 | Keypoint reasoning framework. Keypoint reasoning tries to capture the reasoning relationship between question and the correct answer, which is represented with a reasoning matrix computed by the dot-product of vectors in question and answer. A deep attention strategy consisting of three layers are conducted to extract key points (medical words/concepts) in question and answers.

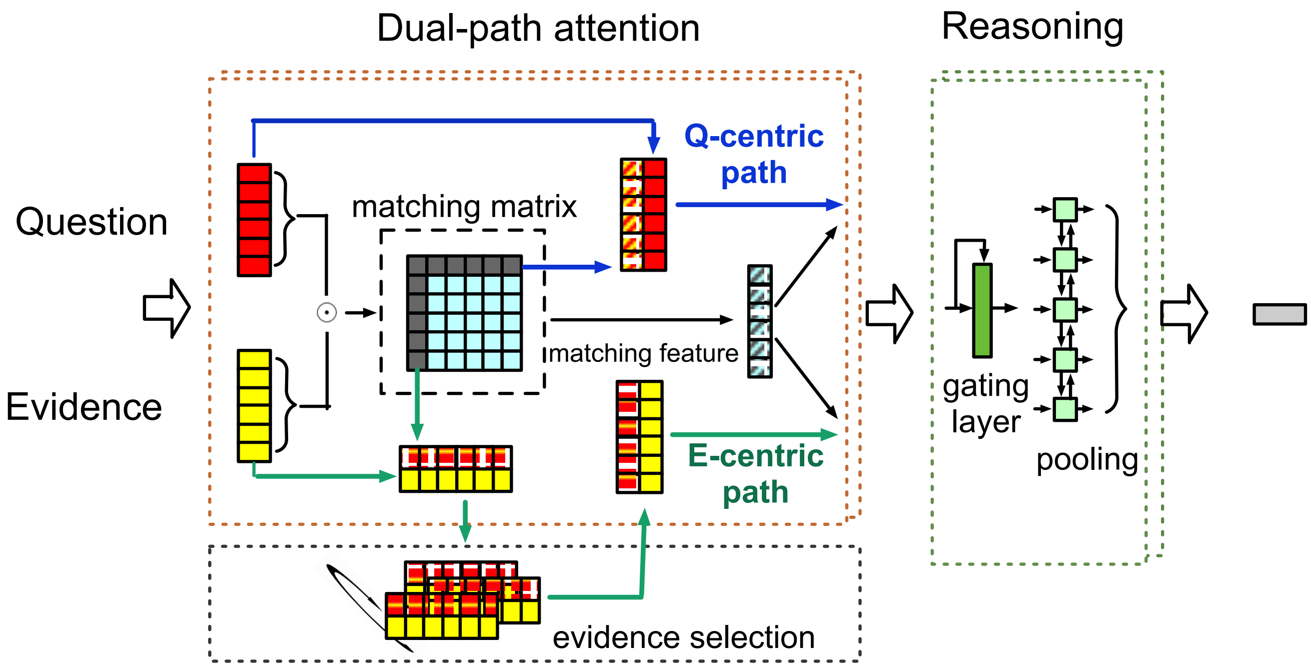

Supplementary Figure 11 | Context reasoning framework. LSTM networks and attention are the main components of context reasoning module. Bi-directional LSTM is used to first convert word embeddings to context vectors. The matching matrix is the dot-product of context vectors in the question and the evidence. Attention is performed row-wise and column-wise on the matching matrix to form question-centric (Q-centric) and evidence-centric (E-centric) representations. The 2 representations are combined with another set of matching features, which are extracted directly from the matching matrix. The reasoning layer takes the combined input, uses a gating layer to weight different contexts, and uses another bi-directional LSTM network to compare the question and the evidence.

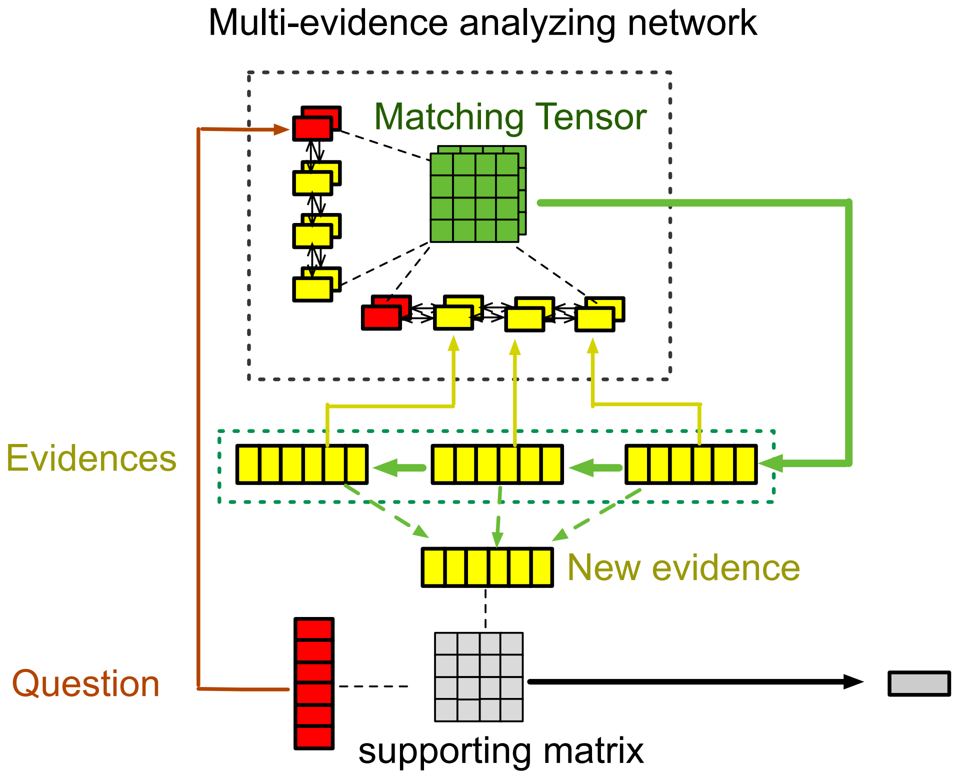

Supplementary Figure 12 | Global reasoning framework. The global reasoning module includes a multi-evidence analyzing network to analyze the semantic relations between the question and a group of evidences. LSTM networks are used to summarize question and each evidence into vector representations. Dot-product of these vectors gives a matching tensor which measures the semantic similarities. Next context representations of a group of evidences are pooled together to assemble a distilled evidence, given the matching tensor. The new evidence contains relevant pieces from all the evidences, and is finally compared with the question to find support of the statement in the question.

Supplementary Table 1 | Clinicians information.

Clinician	Education	Title	Experience (years)
Expert#1	Bachelor	Resident doctor	8
Expert#2	Bachelor	Resident doctor	6
Expert#3	Master	Resident doctor	3
Expert#4	Bachelor	Resident doctor	5

Supplementary Table 2 | Summary of embedding spaces and the corresponding relationships.

Embedding space	Relationship	Description
E_1	R_1	two words are neighboring
E_2	R_2	two words are in the same sentence
E_3	R_3	two words are in the same paragraph
E_4	R_4	two words are in the same section
E_5	R_5	disease and its symptom description
E_6	R_6	disease and its examination description
E_7	R_7	disease and its differential diagnosis description

Supplementary Table 3 | Disease information.

Index	Disease code	Disease name (in Chinese)	Disease name (translated in English)
1	B35.3	脚癣[足癣]	Tinea pedis
2	E11.9	2型糖尿病	Type 2 diabetes
3	E14.9	糖尿病不伴有并发症	Diabetes without complications
4	G47.0	初发性或维持性睡眠障碍［失眠症］	Disorders of initiating and maintaining sleep [insomnia]
5	I10.x	特发性(原发性)高血压	Essential (primary) hypertension
6	I63.9	脑梗死	Cerebral infarction
7	I67.8	脑血管疾病，其他特指的	Other specified cerebrovascular diseases
8	I67.9	脑血管病	Cerebrovascular disease
9	J02.9	急性咽炎	Acute pharyngitis
10	J03.9	急性扁桃体炎	Acute tonsillitis
11	J04.1	急性气管炎	Acute tracheitis
12	J06.9	急性上呼吸道感染	Acute upper respiratory tract infection
13	J20.9	急性支气管炎	Acute bronchitis
14	J30.4	变应性鼻炎	Allergic rhinitis
15	J40.x	支气管炎	Bronchitis
16	J42.x	慢性支气管炎	Chronic bronchitis
17	J44.1	慢性阻塞性肺病伴有急性加重	Chronic obstructive pulmonary disease with acute exacerbation
18	J45.9	哮喘	Asthma
19	J98.4	肺的其他疾患	Other lung diseases
20	K04.0	牙髓炎	Pulpitis
21	K05.2	急性牙周炎	Acute periodontitis
22	K05.3	慢性牙周炎	Chronic periodontitis
23	K12.1	口炎，其他形式的	Other forms of stomatitis
24	K21.0	胃-食管反流性疾病伴有食管炎	Gastric-esophageal reflux disease with esophagitis
25	K27.9	消化性溃疡不伴有出血	Peptic ulcer without haemorrhage
26	K29.7	胃炎	Gastritis
27	K30.x	消化不良	Indigestion
28	K52.9	非感染性胃肠炎和结肠炎	Non-infectious gastroenteritis and colitis

29	K81.9	胆囊炎	Cholecystitis
30	K92.9	消化系统的疾病	Digestive diseases
31	L08.9	皮肤和皮下组织的局部感染	Local infection of skin and subcutaneous tissue
32	L23.9	变应性接触性皮炎	Allergic contact dermatitis
33	L30.9	皮炎	Dermatitis
34	M13.9	关节炎	Arthritis
35	M19.9	关节病	Arthropathy
36	M47.2	神经根型颈椎病	Nerve root cervical spondylosis
37	M47.9	脊椎关节强硬	Spondylosis
38	M51.2	腰椎间盘突出	Lumbar disc herniation
39	M54.3	坐骨神经痛	Sciatica
40	M54.5	下背痛	Lower back pain
41	M75.0	粘连性肩关节囊炎	Adhesive capsulitis of shoulder
42	M81.9	骨质疏松	Osteoporosis
43	N39.0	泌尿道感染	Urinary tract infection
44	N73.9	女性盆腔炎性疾病	Female pelvic inflammatory disease
45	N76.0	急性阴道炎	Acute vaginitis
46	N92.6	月经不规则	Irregular menstruation
47	N95.1	绝经期和女性更年期状态	Menopausal and female climacteric states
48	R42.x	头晕和眩晕	Dizziness
49	Z33.x	妊娠状态	Pregnant state
50	ZDY.0	冠心病	Coronary heart disease
